# The Role of Vitamin D in Respiratory Allergies Prevention. Why the Effect Is so Difficult to Disentangle?

**DOI:** 10.3390/nu12061801

**Published:** 2020-06-17

**Authors:** Hanna Sikorska-Szaflik, Barbara Sozańska

**Affiliations:** 1st Department and Clinic of Paediatrics, Allergology and Cardiology Wrocław Medical University, ul. Chałubińskiego 2a, 50-368 Wrocław, Poland; bsoz@o2.pl

**Keywords:** vitamin D, allergy, asthma, allergic rhinitis, prevention

## Abstract

Asthma and allergic rhinitis are the most common chronic childhood diseases with an increasing prevalence worldwide. There is an urgent need to look for methods of preventing allergic diseases from an early age. The relationship between vitamin D status and allergic diseases has been discussed in several studies recently. 25-hydroxyvitamin D (25(OH)D) is suggested to affect the development and/or severity of asthma and allergic rhinitis. Observational studies have seemed to confirm that vitamin D deficiency may contribute to an increase in allergy and asthma. Following interventional studies, however, have yielded ambiguous results. In this review, we describe recent findings regarding 25(OH)D impact on allergic diseases and provide a systematic analysis of the causes of great variability of the achieved results in different studies.

## 1. Introduction

In recent decades, the incidence of respiratory allergic diseases has increased dramatically [1]. Bronchial asthma and allergic rhinitis (AR) have become the most common chronic diseases in childhood [2,3]. This rapid increase is mainly observed in Western countries. There is an urgent need to search for the methods of prevention of allergic diseases from an early age. Intervention options are sought by attempting to modulate environmental factors and lifestyle that have been identified to promote the risk of allergic diseases. Rural environment, microbiome, exposure to allergens and pollution, but also the impact of changes in diet were considered as the main elements. It has been revealed that many dietary components can influence immunological mechanisms of which we will focus here on the role of 25-hydroxyvitamin D (25(OH)D).

Much attention has been paid to the impact of 25(OH)D on the prevention and treatment of respiratory allergic diseases. The increase in asthma and allergic diseases coincided with vitamin D deficiency depicted in many populations [4]. The inverse relationship has raised the idea of a possible underlying association between these two opposite trends. Further research on the immunomodulatory effect of 25(OH)D in the early stages of immune system development allowed the building of a molecular basis of this hypothesis. Initial observational studies have seemed to confirm that vitamin D deficiency may contribute to an increase in allergy and asthma. Following interventional studies, however, have yielded ambiguous results: from the great hope of preventing asthma and allergic rhinitis with vitamin D supplementation [5] to disappointment [6]. These discrepancies may be influenced by many variables which affect the final effect.

In this article we aimed to present the role of vitamin D in asthma and respiratory allergies prevention with the special attention to discussing the potential reasons of great variability of the achieved results in different surveys. Awareness of these differences may help in planning future research and, as a consequence, in creating explicit practical recommendations for 25(OH)D application.

## 2. Molecular Mechanisms of 25(OH)D Action in Asthma and Allergy Prevention

**Figure 1:**
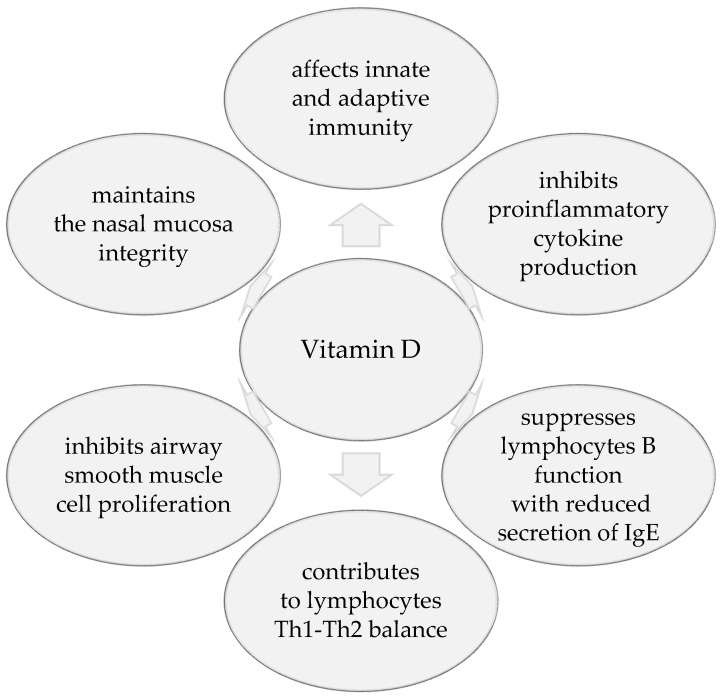
Protective role of vitamin D in respiratory allergic diseases.

Vitamin D is an important modulator of the immune system response and may influence the development of asthma and allergy susceptibility through different mechanisms (Figure 1).

Vitamin D is the general term encompassing both vitamin D2 (ergocalciferol) and vitamin D3 (cholecalciferol). It is formed in 80% of cases from 7-dehydrocholesterol produced in the skin under the influence of UVB radiation, but is also absorbed from the diet in form of vitamin D2 (with plant foods and mushrooms) or vitamin D3 (with fishes, eggs, animal liver, dairy products). Vitamin D, produced in the skin or absorbed from the diet must be activated. It is metabolized first by liver hydroxylases containing cytochrome P450 to 25-hydroxyvitamin D (calcidiol, 25(OH)D). Calcidiol is the major circulating form of vitamin D and is considered the best and most accurate indicator of overall vitamin D status in the organism. Then, in the kidney 25(OH)D is further metabolized to fully active 1 α,25-dihydroxyvitamin D (1 α,25(OH)_2_D, calcitriol). The transforming enzyme, CYP27B1, 1α-hydroxylase, is found not only in the kidneys but also in the extrarenal tissues. Calcidiol after binding to the vitamin D receptor (VDR) is able to initiate or silence gene transcription. Vitamin D is a member of the steroid hormones family and has a nuclear receptor which acts as a ligand-activated transcription factor [7]. Subtle allelic variants of the VDR gene located on chromosome 12-12q13.1 are relatively common in the population. The long arm of chromosome 12 is also a region commonly linked to asthma [8]. VDRs are expressed not only in the tissues such as bones, skin, intestine or kidneys, but also in the brain, eyes, heart muscles, adipose tissue and in almost all cells of the immune system.

Vitamin D exerts complicated and pleiotropic roles far beyond regulation of calcium–phosphorus homeostasis and bone metabolism. It affects both innate and adaptive immunity with immunomodulatory effect. Vitamin D inhibits the expression of Toll-like receptors on monocytes, proinflammatory cytokine production, and induces antimicrobial peptide synthesis. It influences T-cell activation and antigen-presenting cells function [9]. In a murine model of pulmonary eosinophilic inflammation 25(OH)D altered cytokine expression profiles, immunoglobulin E levels, and the pattern of airway eosinophilia during allergen sensitization. It suggests a role of 25(OH)D in the development of allergy and asthma [10]. It contributes to lymphocyte Th1-Th2 balance [9]. Some studies have shown that vitamin D deficiency can lead to the increase in Th2 and reduction in Treg lymphocytes and IL-10 which has been associated with asthma [11]. In other experiments it inhibited macrophage production of IL-12 and suppressed Th1 cytokine production while preserving Th2 cytokine expression, together with IL-4 which promotes an allergic cytokine profile [8]. It has been documented that 25(OH)D decreases proinflammatory cytokine release from peripheral blood mononuclear cells, including lymphocytes T. Inhibition of Th1 cytokine secretion leads to reduced T cell proliferation. The effect of vitamin D on Th2 lymphocytes is less known. Probably it may differ depending on its state of activation—that would explain the conflicting results of different studies. Vitamin D also inhibits B-lymphocyte function with reduced secretion of IgE [12]. Vitamin D action in the immunological system may be dose-dependent. Standard doses may inhibit production of Th1 and Th2 cytokines whereas high doses may even amplify the Th2 responses [13].

Moreover, vitamin D may exert a direct effect in the airways. VDR is present in bronchial smooth muscles cells and vitamin D, interacting through it, can inhibit muscle proliferation. It has been demonstrated that vitamin D can influence growth and survival of cells and, in this way, affects airway remodeling—an important aspect of asthma pathophysiology and treatment. Calcitriol inhibited airway smooth muscle cell proliferation not by inducing apoptosis but by preventing progression of the cell cycle [14]. It may explain the prenatal effect where low levels of 25(OH)D during pregnancy led to developmental changes including reduced lung and airway growth increasing the risk of asthma development. Some studies have shown that 25(OH)D takes part in maintaining the epithelial nasal mucosa integrity, protecting it against environmental allergens in the course of AR [15]. It may also stabilize a mast cell through inhibitory effects on its receptor [16].

Vitamin D affects not only the occurrence but also the course and treatment of asthma. In an experimental study adding 25(OH)D to CD4+, T-cell cultures from steroid resistant patients boosts the response to dexamethasone by enhancing the production of IL-10. Oral administration of 25(OH)D in severe asthmatics inverted steroid resistance through induction of IL10-secreting regulatory T-cells [17].

## 3. Clinical Background—Observational Studies

Many observational surveys focused on the relationship between 25(OH)D level and the incidence or/and the severity of allergic diseases, but they gave conflicting results. In some studies the association between vitamin D deficiency and higher risk of allergic diseases was shown. Hollams et al. in a longitudinal cohort study revealed that vitamin D deficiency may be a cause of increased incidence of asthma and allergy symptoms. Authors checked almost 700 children at the age of 6 years and then at the age of 14 years old. They showed that 25(OH)D levels at ages 6 and 14 were predictive for allergy/asthma outcomes at both ages. Moreover, it was presented that 25(OH)D levels at the age of 6 years were predictive for consequent atopy and asthma-associated phenotypes at the age of 14 years [18]. Additionally, significantly lower levels of 25(OH)D were shown in children with therapy-resistant asthma, than in those with moderate asthma [19]. Interestingly, the authors revealed that longer time spent outdoors in the sun in winter, which favors the production of 25(OH)D, increased the risk of AR [20].

The level of 25(OH) D may also influence the clinical course of asthma. An inverse relationship between child’s 25(OH)D level and severity of asthma attacks, number of exacerbations, intake of inhaled corticosteroids and acceptable disease control was shown [19]. Moreover, studies both in children and adults, showed that vitamin D deficiency has been associated with decreasing pulmonary function expressed as lower mean FEV1 and FVC [21,22]. In a study by Joudi et al. the relationship between the serum 25(OH)D level and response to one-year subcutaneous allergen immunotherapy was found. The higher the patient’s 25(OH)D level, the better the immunotherapy effect that was achieved [23].

As, many of allergies manifestations are presented early in life, prenatal environment might play an important role in asthma and AR development. Vitamin D deficiency is common worldwide, ranging from 45 to 90% in pregnant women and 61–94% in infants [24]. The fetus is completely dependent on 25(OH)D transmission across the placenta from pregnant mother. Feng et al. conducted a study to check the associations between 25(OH)D levels in cord blood or maternal venous blood and risk of offspring’s asthma, wheezing and respiratory tract infections. Low gestational 25(OH)D level influenced the child’s lungs and immune functions and contributed to childhood asthma development [25].

In contrast, there are papers not confirming the considered relationship between vitamin D and allergies. A prospective study revealed no associations among maternal or cord blood 25(OH)D levels in children and atopy, AR and asthma risk outcomes at the age of 5 [26]. There is also much less evidence for the vitamin D impact on asthma clinical course in adults [27]. In a recently conducted meta-analysis, associations between in utero exposure to vitamin D and lower incidence of respiratory tract infections but not reduced risk of wheeze episodes, asthma, atopic eczema, allergic rhinitis, and allergic sensitization were presented [28].

Furthermore, we can find diverse results even within one specific study. Discrepancies may be related to the different ages of the analyzed populations. It was shown that serum levels of 25(OH)D in 4 years old children were inversely correlated with asthma at the ages of 4–8, whereas 25(OH)D concentrations at the age of 8 were positively associated with asthma at that age [29]. In meta-analysis of studies examining the association between 25(OH)D levels and AR, no significant relationship between the cord blood 25(OH)D level and the incidence of AR in children was confirmed. However, current 25(OH)D levels were associated negatively with the prevalence of AR and were lower in patients with AR than in controls in children, but not in adults. The reverse causality cannot be excluded with low 25(OH)D levels caused by AR (AR patients avoid outdoor activities thereby sun exposure and this decreases skin 25(OH)D production) [30]. Further differences in the results are related to patients’ 25(OH)D levels. In Rothers’ birth cohort both low (<50 nmol/L) and high (>100 nmol/L) levels of cord blood 25(OH)D were associated with increased aeroallergen sensitization in children at the age of 5 [31]. Furthermore, Hyppönen showed an ‘U-shaped’ relation between serum 25(OH)D and total IgE in adults. Increase in total IgE concentration was observed at 25(OH)D levels lower than 25 nmol/L and higher than 135 nmol/L [32].

## 4. Clinical Background—Interventional Studies

Promising, but inconclusive results of observational studies became a background for interventional trials on the role of vitamin D supplementation in asthma and allergic diseases prevention and treatment. Many studies confirmed such a relationship. In a trial conducted among children with AR, supplementation of 1000 IU vitamin D daily was associated with several immunomodulatory effects leading to improvements in clinical symptoms [33]. A study in children with asthma showed that, vitamin D supplementation in a dose of 500 IU/day additionally to inhaled corticosteroids, reduced the risk of asthma exacerbation caused by respiratory tract infections [34]. In the research of Arikoglu et al. in children with uncontrolled and well controlled asthma, mean serum 25(OH)D level was significantly lower in children with severe symptoms. Its increase was associated with a lower risk of asthma attacks, independent of age, sex, allergic markers, use of inhaled steroids, BMI, exposure to sun (as a marker of activity level) and serum levels of cathelicidin [35]. Cathelicidin is involved in immune response; its reduced level caused by vitamin D deficiency can create a predisposition to infections in both healthy children and asthmatics. Bunyavanich et al. found a protective effect of vitamin D from food sources (not from supplements) on reducing the risk of AR in children. He studied over 1200 pregnant mother–child pairs and assessed maternal intake of vitamin D in the first and second trimester. Mothers’ serum 25(OH)D levels were also obtained. Higher maternal vitamin D intake during the first and second trimester of pregnancy was associated with approximately 20% lower risk of AR at school age [5].

However, in a Finnish study, supplementation of vitamin D during the first year of life was associated with higher prevalence of AR, atopy, and asthma at the age of 31 years, independently of the dose of the vitamin D supplementation [36].

In the Vitamin D Antenatal Asthma Reduction Trial (VDAART), Litonjua et al. aimed to study whether supplementation would reduce the incidence of asthma diagnosed by a doctor and wheeze episodes reported by the parents during the first 3 years of children’s lives. Authors recruited 881 pregnant women at 10–18 weeks of pregnancy with their or their partner’s asthma/allergies history. Both outcomes in children at age 3 years were rarer in the supplemented mothers’ group, but this result was not significant [37]. In Copenhagen Prospective Studies on Asthma in Childhood 2010 (COPSAC_2010_), women at 24 weeks of pregnancy were recruited and divided into two groups—first was given 2800 IU/day and second 400 IU/day of vitamin D. Maternal supplementation of vitamin D did not reduce child’s symptoms of wheezing at age 3 and did not affect the onset of doctor diagnosed asthma, upper and lower respiratory tract infections, allergic sensitization, and eczema [38]. Interestingly, in combined analysis of these two above trials the supplementation of vitamin D in pregnant women resulted in a significant and clinically important 26% reduced risk of asthma/episodes of recurrent wheezing in the offspring until age 3. This reduced risk was even more pronounced (46%) if 25(OH)D levels achieved at least 30 ng/mL at the beginning of the study [39]. The follow-up results of VDAART cohort revealed that vitamin D supplementation alone in the prenatal period did not affect the development of asthma and wheezing symptoms or both in young children up to 6 years of age [6]. However, it was pointed out that in the same cohort, if younger children aged 1–3 were considered, a lower frequency of wheezing episodes, especially in the first year of life, was observed. It has been concluded than even if vitamin D supplementation cannot prevent asthma in 6 year-olds it still may bring benefits in the form of less persistent forms of wheezing symptoms in infants with allergic family history [40].

## 5. The Role of Vitamin D in Asthma and Respiratory Allergies Prevention: Where do the Different Conclusions Come from?

**Figure 2:**
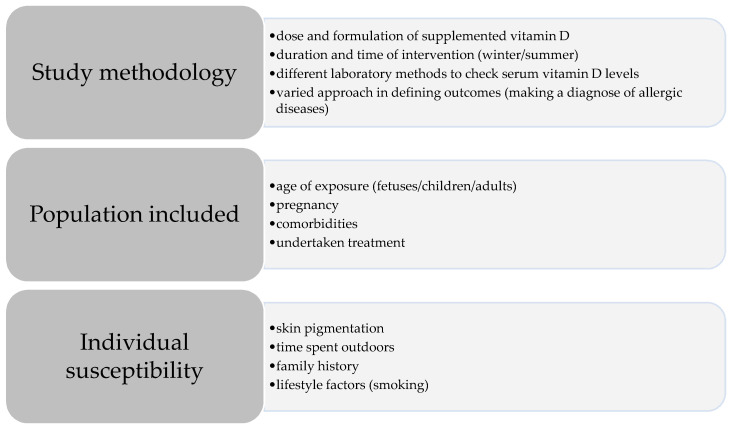
Factors contributing to variability of study findings.

Many clinical and experimental studies suggested vitamin D supplementation as a method of a primary prevention in allergic diseases and asthma. Therefore, the final role of it is still not established and contradictory results have been published. Incompatibilities may arise from different study methodology, different populations included, time of exposure, individual susceptibility, genetic predisposition and many more (Figure 2). Here we discuss some of these aspects.

First, let’s consider methodological disparities. In interventional studies different doses of vitamin D were supplemented, from 400 and 1000 to even 4000 IU/day [33,37]. Higher doses used did not ensure a stronger effect of vitamin D on the allergic prevention. According to the guidelines for optimizing clinical studies of nutrient effects, trials should be based on 25(OH)D levels, not on vitamin D dose [41,42]. Not only doses, but also formulation of vitamin varied. Some authors relied only on measuring the amount of vitamin D consumed with food. This diet dose of vitamin D was not taken into account in many interventional studies with supplementation. Moreover, trials using dietary intake of vitamin D as the marker of its status, may be confounded by patients’ sun exposure and the resulting natural skin production of vitamin D. The initial level of 25(OH)D was not always checked, leading to unclear interpretations of the results in deficient and normal range patients.

Duration and time of intervention differs among the surveys. Some authors decided on short term vitamin D supplementation like Bakhshaee et al. (2 months [43]), while others arranged study for longer periods [33]. In pregnancy it started earlier at 10–18 weeks’ gestation [37] or later at 22–26 weeks [38], which may be too late, as lung development begins before that period. It also influenced duration of supplementation, which might be too short to gain the positive effect. Serum level of 25(OH)D was checked in pregnant mothers/newborn children, outcomes were studied after a few years, not always taking into consideration postnatal supplementation of vitamin D in children, which may have some additive effect. For this reason, making conclusions about the impact of supplementation during pregnancy on the occurrence of allergic diseases in children may be disturbed [6].

Discrepancies may occur because of different laboratory methods used to check serum levels of 25(OH)D and the number of repeated measurements [32]. To minimize this difference, some authors decided to use two methods simultaneously [5]. A number of samples taken in the course of the study varied from one [33] to two, three or even more [31]. This approach, relying on multiple assessments reflects long term status of vitamin D more adequately and allows inferences about its effects. Some samples came from maternal plasma or serum collected during pregnancy; in other studies samples were obtained from umbilical cord blood or both from mothers during pregnancy and children at birth [5,26,28].

Cohort studies designed for a longer period seem to give a better opportunity to precisely check the possible effects of 25(OH)D on the occurrence and course of allergic diseases with time and age. Studies with too short a follow-up period are at risk of bias because of missing the final effect. The chance of positive effects for primary prevention may also change with age of exposure (pregnancy, first year of life or later) and the effect may be pronounced otherwise in different life stages [6].

The discrepancies may be related to a different approach in defining outcomes. Diagnosis of asthma, wheezing or AR may be based on parental reports, self-assessment questionnaires, or physician diagnosis during clinical assessment. Phenotypes and course of disease may look different at different ages. Undertaken treatment also varies and modifies the symptoms [44]. For example, the use of oral steroids can lead to vitamin D deficiency, although the mechanism of this process is complex and not fully understood [45]. Some studies have shown that steroids increase vitamin D metabolism in the kidneys or increase the enzymatic activity of 25(OH)D through glucocorticoid and vitamin D receptors [30].

Patients’ family history of asthma and allergic diseases and comorbidities may be meaningful. In the VDAART study, parent’s asthma and/or AR were mandatory inclusion criterion [37], whereas in other trials populations with or without positive history were included [44]. Studies conducted among patients with a specific disease, e.g., atopic dermatitis are hard to interpret for asthmatics and healthy populations [26].

Vitamin D is partly synthesized in the skin and that process is induced by sun exposure. Hence, not only the dose of vitamin supplemented/consumed with food but also skin pigmentation, time spent outdoors, latitude, and season when the study is conducted (winter/summer) are also essential [46]. Patients with AR may avoid going outside, children with severe asthma would probably stay at home most of the time. Some patients’ lifestyle factors may modify the results of vitamin D studies e.g., smoking or exposure to tobacco smoke is a risk factor for vitamin D deficiency [47].

## 6. Conclusions

A number of trials have shown that vitamin D supplementation may play a role in asthma and allergic diseases prevention, but there are studies not supporting this hypothesis. Taken together, it is not yet possible to definitively state the effect of vitamin D on allergic diseases. Ambiguous conclusions may partly reflect inconsistencies among studies in many aspects. Further research should focus on large, adequately powered human studies, with a standardized approach to supplementation and vitamin D underlying diet, with proper identification of the key factors that influence response and well-defined clinical outcomes at different life stages.
